# Pharmacological Modulation of Anti-Tumor Immunity Induced by Oncolytic Viruses

**DOI:** 10.3389/fonc.2014.00191

**Published:** 2014-07-23

**Authors:** Nicole E. Forbes, Ramya Krishnan, Jean-Simon Diallo

**Affiliations:** ^1^Center for Innovative Cancer Research, Ottawa Hospital Research Institute, Ottawa, ON, Canada; ^2^Faculty of Medicine, University of Ottawa, Ottawa, ON, Canada

**Keywords:** Oncolytic virotherapy, anti-tumor immunity, cancer, combination therapy, pharmacological therapy, chemotherapy, immuno-modulatory therapy

## Abstract

Oncolytic viruses (OVs) not only kill cancer cells by direct lysis but also generate a significant anti-tumor immune response that allows for prolonged cancer control and in some cases cures. How to best stimulate this effect is a subject of intense investigation in the OV field. While pharmacological manipulation of the cellular innate anti-viral immune response has been shown by several groups to improve viral oncolysis and spread, it is increasingly clear that pharmacological agents can also impact the anti-tumor immune response generated by OVs and related tumor vaccination strategies. This review covers recent progress in using pharmacological agents to improve the activity of OVs and their ability to generate robust anti-tumor immune responses.

## Introduction: Oncolytic Viruses: Multi-Mechanistic Biotherapeutics Against Cancer

Oncolytic viruses (OVs) are self-amplifying biotherapeutics that have been selected or engineered to preferentially infect and kill cancer cells. Generated from a multitude of viral species, OVs exploit cancer-associated cellular defects arising from genetic perturbations including mutations and epigenetic reprograming [reviewed in Ref. (1)]. Among others, these cellular defects lead to dysfunctional anti-viral responses and immune evasion, increased cell proliferation and metabolism, and leaky tumor vasculature (2). These characteristics in turn provide a fertile ground for viral replication and subsequent lysis of tumor cells and permit the growth of genetically attenuated OVs that are otherwise harmless to normal cells.

In addition to the direct killing of cancer cells, OVs can also trigger a potent anti-tumor immune response. Infected tumor cells induce the release of pro-inflammatory cytokines and expose both viral and tumor-associated antigens to patrolling immune cells, promoting the differentiation of antigen-presenting cells and T-cell activation (3–5). How much tumor infection and lysis are necessary to trigger these responses remains a topic of debate; however, it is clear that the combination of direct oncolysis and activation of anti-tumor immunity can lead to durable cures in pre-clinical mouse models of cancer.

A number of OVs are currently being evaluated in clinical trials to treat a range of cancer types. For a more comprehensive overview, the reader is invited to consult an excellent review by Russell et al. (6). Of particular note, herpes simplex virus-1 (HSV-1), vaccinia virus, reovirus, and adenovirus-based OV strains have made the most progress toward approval (7–10). Shanghai Sunway Biotech’s oncolytic adenovirus (H101), deleted for the viral E1B gene and thought to target p53 deficient cancer cells, was the first approved OV in China as early as 2005, indicated for head and neck cancers. (11). Profound tumor regression is common following treatment with OVs; for example, durable objective responses were observed in 3/14 patients (hepatocarcinoma, lung cancer, and melanoma) following treatment with vaccinia virus JX-594 in a phase I trial (7). This virus has been deleted for viral thymidine kinase (TK), making it dependent on cellular TK that is overexpressed in cancer cells (7). In addition to the TK deletion that provides tumor selectivity, the virus also expresses granulocyte macrophage colony-stimulating factor (GM-CSF) to stimulate anti-tumor immunity. Most recently, Amgen’s HSV-1-based talimogene laherparepvec (T-VEC) led to 16% durable response in a phase III clinical trial for late-stage melanoma, and it is expected that the company will file for FDA approval in North America in the coming year (12, 13). Like JX-594, T-VEC expresses GM-CSF but has deletions in viral genes ICP34.5 and ICP47 that confer tumor selectivity and promote antigen presentation, respectively (14).

While widespread approval and clinical implementation of oncolytic virotherapy are in the foreseeable future, heterogeneity in clinical response to OVs remains a significant challenge as evidenced from a number of early and late-stage human clinical trials (6, 15, 16). This heterogeneity in response can be attributed to factors that impact OV delivery and spread within tumors, such as pre-existing immunity and remnant tumor anti-viral responses, as well as to a variably immunosuppressive tumor microenvironment that can prevent the generation of an effective anti-tumor immune response. To overcome these challenges, it has been long recognized in the OV field that improvements to therapeutic efficacy either through viral engineering or through combination therapies will be critical (6, 17). In the current review, we will focus on advances in therapeutic strategies employing small-molecule pharmacological agents that ameliorate OV treatment *in vivo* by manipulating the innate and/or adaptive immune response to virus and tumor (summarized in Table 1).

**Table 1:** **Combinations of pharmacological and oncolytic therapies with demonstrated improvements in *in vivo* treatment efficacy**.

Drug	Mechanism of action/molecular target	Reported immunomodulatory effect (systemic immunomodula- tion or specific modulation of anti-viral response	Oncolytic virus	Reference
**CLASSIC CHEMOTHERAPY AGENTS**				
Cyclophosphamide	DNA alkylation	Systemic immunomodulation	HSV	Ikeda et al. (20), Ikeda et al. (21), Wakimoto et al. (22), and Currier et al. (24)
			Adenovirus	Thomas et al. (25), Dhar et al. (26), Cerullo et al. (27), and Hasegawa et al. (28)
			Vaccinia	Lun et al. (29)
			Reovirus	Qiao et al. (30) and Kottke et al. (34)
			Measles	Ungerechts et al. (31) and Ungerechts et al. (32)
Gemcitabine	Nucleoside substitution and inhibition of DNA replication, ribonucleotide reductase inhibitor	Systemic immunomodulation	Adenovirus	Leitner et al. (38), Liu et al. (39), Onimaru et al. (40), Bhattacharyya et al. (41), Cherubini et al. (42), Wang et al. (43), and Kangasniemi et al. (44)
			Parvovirus	Angelova et al. (45)
			Reovirus	Gujar et al. (48)
			VSV	Hastie et al. (49)
			HSV	Watanabe et al. (50) and Esaki et al. (51)
			Vaccinia	Yu et al. (52)
			Myxoma	Wennier et al. (53)
Bortezomib	Proteasome inhibition	Systemic immunomodulation	VSV (VSV-mIFNβ)	Yarde et al. (61)
			Reovirus	Carew et al. (62)
			Adenovirus (hTERT-Ad)	Boozari et al. (63)
Mitoxantrone	Type II topoisomerase inhibition	Systemic immunomodulation	HSV	Workenhe et al. (69)
Irinotecan	Type I topoisomerase inhibition	systemic immunomodulation	HSV	Tyminski et al. (74)
			Sindbis	Granot and Meruelo (75)
Temozolomide	DNA alkylation	Systemic immunomodulation	Adenovirus	Alonso et al. (80), Holzmuller et al. (81), Liikanen et al. (82), and Tobias et al. (83)
			HSV	Aghi et al. (84) and Kanai et al. (85)
**EPIGENETIC MODULATORS**				
Valproic acid	Histone deacetylase inhibition	Specific modulation of anti-viral response	HSV	Otsuki et al. (106)
Trichostatin A	Histone deacetylase inhibition	Specific modulation of anti-viral response	HSV	Liu et al. (105)
			Vaccinia	MacTavish et al. (108)
Entinostat (MS-275)	Histone deacetylase inhibition	Both	VSV	Nguyen et al. (99) and Bridle et al. (109)
5-Azacitidine	DNA methyltransferase inhibition	Specific modulation of anti-viral response	HSV	Okemoto et al. (111)
**PI3K/Akt/mTOR PATHWAY INHIBITORS**				
LY294002	PI3K inhibition	Specific modulation of anti-viral response	HSV	Kanai et al. (116)
Rapamycin	mTORC1 and mTORC2 inhibition	Both	Adenovirus	Jiang et al. (120)
			HSV	Fu et al. (121)
			VSV	Alain et al. (122)
Everolimus (RAD001)	mTORC1 inhibition	Both	Adenovirus	Lukashev et al. (119)
**OTHER**				
Viral sensitizer 1 (VSe1)	Unknown	Specific modulation of anti-viral response	VSV	Diallo et al. (125)
Triptolide	Global transcription inhibition via RNA pol II inhibition	Specific modulation of anti-viral response	VSV	Ben Yebdri et al. (130)
Sunitinib	Receptor tyrosine kinase inhibition	Specific modulation of anti-viral response	VSV	Kottke et al. (87) and Jha et al. (88)
			Reovirus	Kottke et al. (87)
			Vaccinia	Hou et al. (89)
Ipilimumab	CTLA-4 inhibition	Systemic immunomodulation	NDV	Zamarin et al. (138)

*Numerous studies have shown that combining oncolytic virotherapy and pharmacological therapy leads to improved outcomes *in vivo*. This table summarizes these reports, presenting the small molecule used in the study, its main mechanism of action or molecular target, its reported immuno-modulatory effect(s), and type of oncolytic virus used. Abbreviations: HSV, herpes simplex virus; VSV, vesicular stomatitis virus; mIFNβ, murine interferon beta; hTERT-Ad, human telomerase reverse transcriptase promoter-regulated adenovirus; PI3K, phosphoinositide 3-kinase; mTOR, mammalian target of rapamycin; mTORC1, mammalian target of rapamycin complex 1; mTORC2, mammalian target of rapamycin complex 2; CTLA-4, cytotoxic T-lymphocyte antigen 4*.

## Standard Chemotherapeutic Drugs that Boost OV Activity through Systemic Effects on Immune Cells and the Immune Response

Most cancer patients with advanced disease will be subjected to some form of chemotherapy. This will largely depend on the type of cancer and other salient pathophysiological characteristics. Given that most patients enrolled in clinical trials to test the efficacy of OVs suffer from advanced disease (7), a natural trend in the OV field has been to test OVs in combination with chemotherapeutics that are currently the standard of care. Classic chemotherapy drugs typically capitalize on the fact that cancer cells are continuously replicating unlike most normal cells (18). However, some normal cell types have higher replication rates, leading to significant off-target effects. Hematopoietic cells among others can be affected and this can lead to systemic immunosuppression (discussed below). While the evaluation of chemotherapeutic drugs in the context of OV therapy has been fairly empirical for the most part, their immunosuppressive effects can inherently complement OV activity by increasing OV spread within tumor beds and/or increasing anti-tumor immune responses. The following sections provide an overview of classic chemotherapy drugs that have been evaluated in combination with OVs focusing on their anti-cancer mechanism of action, examples of OVs with which they have been tested, and the mechanism by which these agents suppress immunity and co-operate with OVs to improve therapeutic outcomes.

### Cyclophosphamide

Cyclophosphamide (CPA) is a nitrogen mustard alkylating agent that leads to cross-linking of nucleotides. Its active metabolite, phosphoramide mustard, interferes with DNA replication by forming guanine-to-guanine intra-strand and inter-strand crosslinks (19). Aldehyde dehydrogenase (ALDH) catalyzes the conversion of the immediate precursor of phosphoramide mustard, aldophosphamide, to an inactive metabolite. Normal cells, for example intestinal epithelial cells and bone marrow stem cells, have a high level of ALDH, protecting them from the effects of CPA’s toxic metabolites. In contrast, some lymphocytes have a lower level of ALDH, which makes them more susceptible to the effects of CPA. CPA has been used in combination with several OVs including HSV-1 (20–24), adenovirus (25–28), vaccinia (29), reovirus (30), measles (31–33), and vesicular stomatitis virus (VSV) (33), leading to improved anti-tumor activity *in vivo*. Several studies suggest that CPA can be efficacious in combination with OVs by preventing immune-mediated viral neutralization through inhibiting or delaying the rise of neutralizing antibodies and depleting anti-viral immune cells including natural killer (NK) cells, monocytes, macrophages, and lymphocytes (20, 22, 23, 25, 26). For example, one study showed that CPA inhibits tumor infiltration of innate phagocytes (macrophages, microglia, and NK cells) following HSV treatment in a syngeneic rat glioma model, leading to increased viral persistence and improved overall efficacy (23). Other studies suggest CPA can also enhance the generation of anti-tumor immunity by inhibiting regulatory T-cells (Tregs) (27, 34). Results from a first in-human clinical trial using Ad-GM-CSF (CGTG-102) to treat solid tumors suggest that metronomic dosing of CPA decreases Tregs without compromising the induction of anti-tumor T-cell responses. This was found to be associated with increased cytotoxic T-cell responses and the induction of Th1 type immunity in most patients. The best progression-free survival and overall patient survival rates were seen with the combination of metronomic CPA and intratumoral infection of adenovirus (27).

### Gemcitabine

Gemcitabine is a fluorinated deoxycytidine nucleoside analog. Incorporation of this analog into DNA prevents further addition of nucleosides during DNA polymerization and thereby halts DNA replication and cell division. Gemcitabine also binds irreversibly to the active site of ribonucleotide reductase. As a result, nucleotide production is halted and DNA replication ceases, leading to apoptosis in rapidly dividing cells [reviewed in Ref. (35)]. While gemcitabine can decrease neutralizing antibodies similar to CPA (36), it is thought to promote anti-tumor immune responses by off-target elimination of myeloid derived stem cells (MDSCs), which suppress T-cell responses. Gemcitabine treatment thereby increases the activity of CD4+ and CD8+ T-cells that recognize tumor antigens (37). This drug has been shown to increase the anti-tumor activity of a wide array of OVs including adenovirus (38–44), parvovirus (45, 46), reovirus (47, 48), VSV (49), HSV (50, 51), vaccinia (52), and myxoma virus (53). In the latter example, the anti-cancer activity of oncolytic myxoma virus was improved using gemcitabine in disseminated pancreatic cancer murine models (53). Interestingly, no sensitization occurred in immunocompromised mice, supporting the requirement for a virus-triggered anti-tumor immune response in mediating the combination effect. The combination of gemcitabine and reovirus was recently evaluated in a phase I clinical trial and while anti-tumor immune responses were not measured, neutralizing antibodies against reovirus were decreased by gemcitabine treatment. In this study, 80% of evaluable patients showed either partial response or stable disease (36).

### Bortezomib

Bortezomib is a proteasome inhibitor approved to treat multiple myeloma and mantle cell lymphoma. It reversibly binds the catalytic site of the 26S proteasome with high affinity and specificity (54). Bortezomib has been shown to inhibit NF-κB by preventing degradation of IκB-α in some cell types (55) although the opposite effect has also been observed (56). Other mechanisms of action by which bortezomib may kill cancer cells are through ER-stress and activation of the unfolded protein response (UPR) (57) and triggering apoptosis by preventing the degradation of pro-apoptotic proteins (56, 58). Some studies have shown that treatment of cancer cells using bortezomib increases surface expression of Hsp90 and Hsp60 in cancer cells leading to their more effective phagocytosis by dendritic cells (DCs), improving tumor vaccine effects (59). Bortezomib-treated mice also exhibit increased DC maturation and phagocytic potential (59). On the other hand, one study found that bortezomib treatment leads to apoptosis of allo-reactive CD4+ T-cells. Thus the net result on anti-cancer and anti-viral immune responses is likely context-dependent (60).

Bortezomib has been tested in combination with oncolytic VSV (61), reovirus (62), and adenovirus (63). Using VSV-mIFNβ, combined treatment with bortezomib was inhibitory to virus replication in myeloma cells *in vitro* but led to improved therapeutic efficacy compared to single treatments in syngeneic murine myeloma models (61). Given no observed effect on tumor viral load, this suggests bortezomib likely increases virus-induced cell death and/or potentiates the anti-tumor response mediated by the virus. Supporting the former, in combination with the oncolytic adenovirus hTERT-Ad, bortezomib enhanced infection-induced ER-stress and activated the UPR and UPR-associated apoptotic cell death *in vitro* (63). In subcutaneous hepatocellular carcinoma (HCC) mouse models, bortezomib refocused the immune response toward tumor-associated antigens by inhibiting immune recognition of the virus. This allowed for a reduction in viral dose in the combination therapy while maintaining similar efficacy. It was further demonstrated that bortezomib’s efficacy is dependent upon a functional CD8+ T-cell response, as no response was seen *in vivo* upon depletion of CD8+ T-cells.

### Mitoxantrone

Mitoxantrone is a type II topoisomerase inhibitor and a DNA intercalating agent. Thus, it disrupts DNA synthesis and DNA repair in both healthy cells and cancer cells (64). Mitoxantrone was initially developed for treatment of cancer and has been notably approved to treat leukemia and prostate cancer. However, due to its immunosuppressive effects, mitoxantrone was also approved for the treatment of multiple sclerosis over a decade ago. Similar to other immunosuppressive chemotherapies, its activity can be attributed to its effects on proliferating immune cells, but it also has additional effects on antigen-presenting cells and enhances suppressor T-cell functions. Mitoxantrone treatment notably reduces the secretion of pro-inflammatory cytokines such as IL-2, interferon-γ (IFN-γ), and tumor necrosis factor alpha (65–68). This drug has been tested in combination with oncolytic HSV-1 in syngeneic murine breast tumor models (69) but only *in vitro* with adenovirus in prostate cancer cells (70–72). In the case of the HSV-1 ICP0 null OV KM100, mitoxantrone was found to induce immunogenic cell death and whereas no enhanced cell killing was observed *in vitro*, the combination treatment improved survival compared to single treatments in a Her2/neu TUBO-derived syngeneic murine tumor model. This effect was associated with increased intratumoral infiltration of neutrophils and tumor antigen-specific CD8+ T-cells. It was also observed that CD8+ and CD4+ T-cells as well as Ly6G+ neutrophils were important in mediating the improved anti-tumor efficacy.

### Irinotecan

Irinotecan or more accurately its active metabolite SN-38 inhibits topoisomerase I leading to a blockade in DNA replication and transcription. It is mainly used in colon cancer as part of a regimen known as FOLFIRI, which also includes folinic acid and 5-fluorouracil. This course of therapy has been found to reduce the number of Tregs in colorectal cancer patients with minimal impact on total lymphocyte and CD4+ T-cells counts (73). Few studies have used irinotecan in combination with OVs *in vivo*. One study showed that HSV-1 expressing CYP2B1, which converts irinotecan into SN-38, leads to improved survival in combination with irinotecan as compared to virus or drug alone in an immunodeficient mouse glioma model (74). While potential immunological effects were not assessed, a likely contributor to the effect of combination therapy is the increased conversion of irinotecan to active SN-38 due to the expression of CYP2B1 by the virus. Another study used oncolytic Sindbis to treat immunodeficient mice bearing human ovarian tumors (75). In this model irinotecan improved the oncolytic efficacy of Sindbis and this effect required NK cells.

### Temozolomide

Temozolomide (TMZ) is an alkylating agent that leads to alkylation/methylation of DNA and has demonstrated clinical benefits in patients with glioblastoma (GBM) (76) and advanced metastatic melanoma (77). At higher doses, TMZ can be myeloablative and in these conditions, CD4+ and CD8+ T-cells, as well as Tregs are markedly reduced. Vaccination using an anti-tumor peptide vaccine following TMZ-induced myeloablation leads to improved CD8+ T-cell anti-tumor responses and prolongs survival in a murine model of established intracerebral tumors (78). However, Treg depletion has also been observed following low-dose TMZ in rats (79). Oncolytic adenovirus (80–83) and HSV (84, 85) have been tested *in vivo* in combination with TMZ, albeit immune effects have not been systematically explored. In one study with Ad5/3-D24-GM-CSF ± low-dose CPA (to reduce Tregs), treatment with TMZ increased tumor cell autophagy, anti-tumor immunity, and ultimately reduced tumor burden in murine models of xenogeneic prostate cancer (82). When used in chemotherapy-refractory patients, adenovirus infusion followed by TMZ treatment was found to increase tumor-specific T-cells and immunogenic cell death as well as overall survival compared to adenovirus treatment alone.

### Sunitinib

Sunitinib is an oral, small-molecule, and multi-targeted receptor tyrosine kinase (RTK) inhibitor that was approved by the FDA for the treatment of metastatic renal cell carcinoma (RCC) and gastrointestinal stromal tumors (GIST) in 2006. Since then it has also been approved for use in neuroendocrine pancreatic cancer. Sunitinib inhibits cellular signaling by targeting multiple RTKs. These include platelet-derived growth factor receptors (PDGF-R) and vascular endothelial growth factor receptors (VEGF-R). Sunitinib also inhibits KIT (CD117), the RTK that drives the majority of GISTs. In addition, sunitinib inhibits other RTKs including RET, CSF-1R, and FLT3. Sunitinib has been recently shown to have additional off-target effects that block effector proteins of the IFN signaling pathway such as RNaseL and PKR (86).

Sunitinib has been evaluated in combination with VSV (87, 88), reovirus (87), and vaccinia virus (89). In the context of VSV oncovirotherapy, sunitinib decreased phosphorylation of the PKR substrate eIF2-α, leading to increased viral titers *in vitro*. Quite remarkably, combination therapy resulted in complete and sustained tumor regression in several immunodeficient and immunocompetent mouse tumor models (88). However, sunitinib may have additional effects on the infectivity of tumor vasculature. One study used sunitinib to transiently inhibit VEGF signaling, creating a “VEGF burst” upon treatment recovery. In combination with oncolytic VSV and reovirus, this led to increased viral infection and endothelial cell lysis as well as virus spread from blood vessels to cancerous tissues (87). A recent study looked at the combined effect of sunitinib and oncolytic vaccinia virus in syngeneic kidney and breast cancer mouse models, and found the combined treatment led to the most dramatic tumor reduction. Infection of tumors with oncolytic vaccinia as a monotherapy led to decreased VEGF expression (89), in line with the observation that vaccinia induces tumor vascular shutdown in both murine tumor models and in patients (90–92). Thereby, the combination effect in this study was attributed to enhanced tumor devascularization, although other potential effects of sunitinib on the cellular anti-viral response cannot be ruled out.

## Drugs That Epigenetically Reprogram Immune Responses to Enhance OV Therapy

Epigenetic changes in gene regulation and expression can lead to phenotypic heterogeneity in genetically identical cell populations. Through reversible modifications to DNA and chromatin structures by enzymes targeting DNA, histones, and the distribution pattern of nucleosomes, the ability of transcriptional factors to access their respective promoters can be deeply altered (93). Not surprisingly, many enzymes that are involved in epigenetic regulation are deregulated in cancer and manipulation of the cancer epigenome using small molecules has been explored successfully as a treatment modality for cancer. As will be discussed in the following sub-sections, modification of the cancer epigenome has also proven beneficial to improve oncolytic virotherapy through effects on the cellular anti-viral response, the anti-tumor immune response, and even viral gene expression [for a more extensive review, refer to Ref. (1)].

### HDAC inhibitors

Transformed cells often have defective IFN signaling pathways due to the cytokine’s ability to suppress cellular proliferation and stimulate immune responses, both of which cancer cells must bypass in order to evolve to full-blown malignancies (94–96). Indeed, it has been estimated that roughly three quarters of tumor cell lines within the NCI60 panel have defective IFN responses (97). Numerous reports have attributed dysfunctional IFN pathways in tumors to epigenetic silencing including DNA promoter hypermethylation and transcriptionally suppressive histone modifications [reviewed in Ref. (1)]. The extent to which interferon-stimulated genes (ISGs), the effector arsenal of the IFN-mediated anti-viral response, are epigenetically silenced can lead to differences in the sensitivity to virus infection (98–102). Importantly, transcriptional activation of ISGs has been shown to require histone deacetylase (HDAC) activity (103), which has spawned the evaluation of HDAC inhibitors (HDIs) in combination with several OVs.

HDAC inhibitors including valproic acid (VPA), trichostatin A (TSA), suberoylanilide hydroxamic acid (SAHA), and MS-275 have all been used in the context of OV therapy to effectively “reprogram” IFN-responsive tumors to become permissive to OV infection. HDIs such as VPA and TSA were found to enhance HSV oncolysis in oral squamous carcinoma cells (SCC) (104) and glioma tumors (105–107). In one report, this was attributed to an inhibition of virally induced ISG expression, even in the presence of exogenously added IFNβ (106). The result of HDI/HSV combination therapy led to prolonged survival in several murine tumor models (105, 106). TSA also enhanced the oncolytic capacity of vaccinia virus, where the two agents synergistically increased cell killing *in vitro* in several cancer cell lines and the combination therapy led to improved survival responses in syngeneic lung metastasis and subcutaneous colorectal carcinoma mouse models (108).

Similarly, MS-275 (entinostat), SAHA (vorinostat), and other HDIs robustly sensitized resistant cells to VSV-mediated oncolysis by suppressing transcription of IFNβ and ISGs, increasing viral titers, and increasing cancer cell death. This potent synergy was cancer cell-specific and led to delayed tumor progression in xenograft models and improved viral spread within tumors in a syngeneic metastatic breast cancer model (99). While only evaluated *in vitro* in this study, HDI treatment of several cancer cell lines increased spreading of vaccinia and Semliki Forest viruses as well. This activity was ultimately linked to HDI-elicited dampening of the response to IFN (99).

In addition to the effects of HDIs on the response to IFN, evidence suggests HDIs can have additional immuno-modulatory properties. Particularly striking effects of HDIs have been observed in the context of a heterologous oncolytic prime-boost strategy, where mice with syngeneic B16 melanoma brain tumors were first primed with an oncolytic adenovirus expressing the tumor-associated antigen dopachrome tautomerase (hDCT, overexpressed in B16) then treated with oncolytic VSV expressing hDCT. MS-275 given along with VSV-hDCT potentiated the anti-tumor response to hDCT while suppressing the adaptive anti-viral response, ultimately redirecting the immune response toward the tumor. As a result, efficacy was dramatically improved, where the majority of mice given MS-275 in the prime-boost regime experienced long-lasting (>200 day) cures, compared to 100% mortality before day 50 in the mice given the same therapy minus MS-275 (109). In this study, it was also shown that MS-275 reduced virus neutralizing antibodies and memory CD8+ T-cells while maintaining prime-induced levels of humoral and cellular immunity against the tumor antigen (109).

### 5-AZA

DNA methylation and histone modifications are highly interdependent epigenetic processes (110). In addition to histone acetylation-mediated gene silencing, ISGs and other genes implicit in the IFN-mediated anti-viral response are often silenced in cancers by DNA hypermethylation at CpG islands in their promoter region [reviewed in Ref. (1)]. In addition to cellular genes, viral genomes can also be susceptible to direct epigenetic silencing. For example, oncolytic HSV rQNestin34.5 is transcriptionally silenced upon infection of glioma cells, due to increased DNA methylation levels at the virally encoded mammalian Nestin promoter (111). As such, some groups have investigated using OVs in combination with 5-AZA-2′-deoxycytidine (5-AZA): a DNA methyltransferase inhibitor that prevents DNA methylation and allows silenced DNA to regain accessibility to transcription factors. In the case of oncolytic HSV rQNestin34.5, treatment with 5-AZA was sufficient to de-repress transcription under control of the Nestin promoter, allowing viral gene expression, increased viral replication, and HSV-mediated glioma cell killing. This translated to increased survival in glioma bearing mice treated with both 5-AZA and the OV, compared to either treatment administered alone (111). However, it is interesting to mention that in the same study, VPA an HDAC inhibitor was sufficient to drive down DNA methylation at the Nestin promoter *in vitro* in infected glioma cells, highlighting the closely interrelated impact of DNA methylation and histone modification (111).

## PI3K/Akt/mTOR Pathway Inhibitors

The phosphoinositide 3-kinase (PI3K) pathway is critical to cell survival/apoptosis signaling in response to stress. Genetic mutations in the P13K pathway frequently occur in cancers resulting in dysfunctional apoptotic responses and pro-survival signaling (112). Various growth hormones and stress signals including IFN-α activate PI3K, which triggers a signaling cascade leading to Akt phosphorylation (112, 113). This activates the kinase, which then phosphorylates a number of cellular factors involved in cell survival and proliferation such as NF-κB, which is also involved in inducing the type I IFN cascade.

Several PI3K pathway inhibitors including GDC-0941 and NVP-BEZ235 are currently being clinically evaluated for the treatment of cancer (114). Both GDC-0941 and LY294002, a common PI3K inhibitor chemical probe, inhibit PI3K activity via competitive inhibition of an ATP binding site on the p85α subunit (115). The PI3K inhibitors LY294002, GDC-0941, BEZ235, as well as the Akt inhibitor tricibine, acted synergistically with oncolytic HSV MG18L to induce apoptosis in glioma cell lines *in vitro* in a cancer cell-specific manner. Remarkably, combination therapy resulted in durable cures in mice bearing glioblastoma multiforme (GBM) tumors, surpassing the efficacy of either therapy administered alone (116). Recent findings also indicated LY294002 increased killing of multiple myeloma cells *in vitro* triggered by the oncolytic adenovirus ZD55-TRAIL (117).

Mammalian target of rapamycin (mTOR), a master regulator of cellular translation, is downstream of PI3K and Akt signaling. Indeed, both GDC-0941 and NVP-BEZ235, a PI3K inhibitor developed by Novartis, have been reported to inhibit mTOR as well as PI3K (114). While mTOR controls translation of a host of cellular mRNAs and can also impact translation of viral proteins, evidence suggests it can control the anti-viral response by regulating translation of IFN and other key mediators of the anti-viral response such as IRF-7 (118). The mTOR inhibitor rapamycin, a well-known immunosuppressant, has been tested in combination with several OVs including oncolytic adenovirus (119, 120), HSV (121), VSV (122), and myxoma (123, 124). Treatment with rapamycin or closely related mTOR inhibitors such as everolimus (RAD001) has been reported to suppress the adaptive immune response to OVs by reducing levels of antibodies generated against the viruses (120), improving OV activity in several rodent models of cancer (119–121). In one study, enhancement of OV activity was also observed *in vitro* following treatment with rapamycin (121). This may be due to the impact of rapamycin on the IFN response as determined from another study where rapamycin was shown to reduce levels of VSV-induced IFN in rats, improving VSV efficacy in an aggressive rat glioma model (122). Interestingly, oncolytic myxoma is enhanced by rapamycin in normally resistant human tumor cells *in vitro*; however, the mechanism by which this occurs is thought to be due to rapamycin-induced increases in Akt kinase levels optimal for sustaining myxoma replication (123).

## Other Promising Immuno-modulatory OV-Enhancing Drugs

### Novel viral sensitizers

The paragraphs above have shown countless examples of empirically or rationally selected combination therapeutic approaches aiming to improve the activity of OVs using well-characterized chemotherapeutics and signaling pathway inhibitors. A high-throughput screen was performed in an effort to expand this approach in an unbiased manner to identify previously uncharacterized small molecules that enhance OV activity. This screen was performed using oncolytic VSVΔM51 in the resistant murine breast cancer cell line 4T1 (125). Several molecules were identified as novel “viral sensitizers” (VSes) that were capable of boosting VSV replication and spread *in vitro*. One of these compounds, VSe1, boosted VSVΔM51 replication by up to 1000-fold, and was found to synergistically increase tumor cell killing. The mode of action of VSe1 is not fully understood but at a minimum it involves disruption of the IFN response. More specifically, ISGs typically triggered upon VSV infection remained silenced in cells pre-treated with VSe1 (125). When used as a combination therapy to treat an aggressive mouse colon carcinoma model refractory to VSVΔM51, VSe1 potentiated OV activity leading to delayed tumor progression in the context of the combination treatment, while either VSVΔM51 or VSe1 alone had no appreciable anti-cancer effects (125).

### Triptolide

Triptolide (TPL) is a naturally derived component of the Chinese herb *Tripterygium wilfordii* and has been used for centuries as an anti-inflammatory remedy that has also been found to have anti-cancer properties (126–128). TPL is known to be a global transcription inhibitor and has multiple effects including the inhibition of RNA polymerase II and the expression of genes involved in apoptosis and NFκB signaling (129). A recent report found that TPL also suppresses IFN signaling downstream of IRF3 (130). When combined with oncolytic VSV both *in vitro* in VSV-resistant tumor cells and *in vivo* in an aggressive mouse GBM tumor model, the two therapies synergistically improved tumor-specific virus replication leading prolonged survival and delayed tumor progression compared to either therapy given alone (130).

### Jak kinase inhibitors

Ruxolitinib (Jakafi) is a Jak1/2 kinase inhibitor (131) approved in 2011 for the treatment of myelofibrosis (132). Patients with myeloproliferative neoplasms often possess an activating mutation in the gene encoding *Jak2* (133), resulting in aberrant inflammatory cytokine release and splenomegaly. Treatment with ruxolitinib, while not targeting the genetic determinant of the neoplasm, led to profound resolution of severe symptoms in human trials to treat myelofibrosis (splenomegaly, weight loss, fatigue), and this clinical efficacy was associated with a potent reduction in inflammatory cytokine levels (134). Given that Jak1 is required for type I IFN signaling and induction of ISGs, Jak1 inhibitors have the potential to benefit OV therapy in IFN-responsive tumors. Both ruxolitinib and Jak inhibitor 1 were sufficient to sensitize VSV-resistant squamous cell carcinoma cells *in vitro* to VSV infection, and this sensitization was associated with marked decreases in ISG expression (135). Pre-treatment with the Jak inhibitor 1 also sensitized sarcoma and bladder carcinoma cells to VSV infection *in vitro* (136).

### Checkpoint inhibitors

Targeting T-cell inhibitory check point molecules, including the T-cell inhibitory receptor cytotoxic T-lymphocyte antigen 4 (CTLA-4) and programed cell death 1 (PD1), is a relatively new therapeutic approach to cancer therapy. During normal immune responses, T-cell checkpoint receptors such as PD1 and CTLA-4 prevent overactive T-cell responses, which can lead to harmful tissue damage. However in cancers, tumor infiltrating T-cells are often inhibited by both PD1 and CTLA-4 stimulation. As a result, T-cell anergy is a major barrier to immune-mediated tumor recognition and clearance. Given the ability of OVs to stimulate an anti-tumor immune response, combining OV with checkpoint inhibitors has emerged as a logical combination approach. While several groups are currently working on this approach, published studies to date have focused on ipilimumab, an anti-CTLA-4 antibody approved to treat melanoma in 2011. By targeting CTLA-4, ipilimumab blocks interaction with its ligands, CD80/CD86, leading to increases in T-cell mediated anti-tumor responses. Anti-CTLA-4 antibodies have been used in combination with oncolytic parvovirus *in vitro* (137) and Newcastle disease virus (NDV) *in vivo* to treat murine B16 melanoma (138). Remarkably, the combination therapy of NDV and anti-CTLA-4 led to nearly 70% cures in a B16 melanoma mouse model compared to 20% cures for anti-CTLA-4 antibody alone and no effect of the OV on its own (138). Notably, NDV complemented with anti-CTLA-4 led to an increase in the infiltration of activated CD8+ and CD4+ T-cells and a reduction in Tregs.

## Conclusion

Successful therapy using OVs will ultimately depend on effectively navigating the delicate balance between the anti-viral response and the anti-tumor immune response such as to minimize the former in the short term and maximize the latter in the long term. As outlined above, several approved drugs and novel small molecules can be effective tools to dampen the innate and adaptive anti-viral responses, increase the anti-tumor immune response, or both. However, given the close interplay between the cellular anti-viral response and the adaptive immune response that is required for prolonged tumor control, OV/drug scheduling is likely to be critical. To this end, it is probable that the combination of some of the agents described above may allow for additional flexibility and more effective therapy. For example, one can easily foresee first using a drug that specifically dampens the cellular antiviral to permit robust OV replication followed with another that promotes the generation of an anti-tumor response. However, given the efficacy of each approach is undoubtedly both context-dependent (e.g., tumor type and tumor site) and OV-dependent, more pre-clinical and clinical studies will be necessary to identify winning combinations that can maximize the potential for curing cancers in a clinical context.

While many studies demonstrate therapeutic benefit of combination therapies at least in animal models, we can perceive a deficit in regards to systematic head-to-head comparisons of different combination therapies coupling OVs and the immune-modulatory drugs reviewed above. While such a feat may prove daunting experimentally, this exercise seems warranted and necessary to delineate a more educated choice of combination therapies to push forward into clinical trials. One clear trend overall is that evaluation of promising combination therapies with novel immuno-modulatory agents seems to stop at the pre-clinical level. There are likely several factors that contribute to this. For example, companies developing novel small molecules may be reluctant to explore combinations with OVs that are still relatively novel themselves. Similarly, novel small molecules need to be validated clinically, which complicates clinical trial design and adds additional risk from the perspective of those spearheading clinical translation of OVs. This is particularly challenging for novel small molecules such as VSe1, which have been selected for the sole purpose of enhancing OV activity (125). This type of small-molecule/OV co-development can only be reasonably achieved by pharmaceutical companies that have experience in developing both small-molecule and biological therapies separately. Hence, from a clinical perspective, it is likely that the combination of OV therapy with a chemotherapy drug that is part of current standard of care would be the easiest to implement as demonstrated with the combination of oncolytic adenovirus and CPA (27). With promising results emerging from the clinic showing benefits combining OVs with traditional chemotherapy drugs, and as pharmaceutical companies such as Amgen begin to take heed of the potential of OV therapy for the treatment of cancer, clinical evaluation of some of the more novel OV-synergizing compounds seems likely in the near future as a means to overcome heterogeneity in clinical response.

## Conflict of Interest Statement

The authors declare that the research was conducted in the absence of any commercial or financial relationships that could be construed as a potential conflict of interest.
